# Patient safety in non-conveyance within prehospital emergency medical services: a register-based study

**DOI:** 10.1007/s11739-025-03980-w

**Published:** 2025-05-22

**Authors:** Frederikke Amalie Møller, Martin Faurholdt Gude, Rasmus Østergaard Nielsen, Tine Bennedsen Gehrt

**Affiliations:** 1 Department of Research and Development, Prehospital Emergency Medical Services, Central Denmark Region, Brendstrupgårdsvej 7, 2. th., 8200 Aarhus, Denmark; 2 Department of Clinical Medicine, Aarhus University, Aarhus, Denmark; 3 Department of Public Health, Aarhus University, Aarhus, Denmark; 4 Research Unit for General Practice, Aarhus, Denmark

**Keywords:** Emergency medical services, Non-conveyance, Treat and release, Patient safety

## Abstract

**Objectives:**

This study investigated the patient safety of prehospital Emergency Medical Service (EMS) non-conveyance decisions by examining EMS reassessments on-scene, hospital admission, and mortality within 48 h among non-conveyed patients. Secondarily, we described proportions of patients within each dispatch code needing reassessment and hospital admission and explored predictors of these outcomes.

**Methods:**

This register-based study included all EMS assignments in the Central Denmark Region from January 1st, 2022, to December 31st, 2023, resulting in EMS-initiated non-conveyance. We estimated the proportion of patients reassessed on-scene by EMS providers, admitted to hospital or deceased within 48 h. Predictors of reassessment and hospital admission were explored using regression models.

**Results:**

During the 2-year period, 17.402 patients were non-conveyed. Among these, 4.70% (95% CI: 4.40–5.03%) were reassessed by EMS providers, 4.92% (95% CI: 4.60–5.25%) were admitted to the hospital within 48 h, and 14 patients died within 48 h (0.08%, 95% CI: 0.04–0.13%). Patients with ‘ear, nose and throat’ complaints had a high risk of needing EMS reassessment (17.6%, 95% CI: 11.5–25.2%) and hospital admission (16.0%, 95% CI: 10.2–23.5%). Furthermore, the risk was high in patients with ‘seizures’, ‘non-traumatic bleeding’ and ‘psychiatry/suicidal ideation’. Male sex, older age, and abnormal vital signs were identified as predictors.

**Conclusions:**

Within 48 h, mortality was low and few non-conveyed patients were reassessed by the EMS and admitted to the hospital. This suggests that non-conveyance is generally a safe practice. However, caution is needed when considering males, older patients, those with specific complaints, and abnormal vital signs.

**Supplementary Information:**

The online version contains supplementary material available at 10.1007/s11739-025-03980-w.

## Introduction

Over the past decades, there has been an increase in the use of prehospital emergency medical services (EMS) and emergency departments (EDs) [1–3]. In Denmark, the number of EMS incidents increased from 24.3 per 1000 inhabitants in 2007 to 40.2 per 1000 inhabitants in 2014 [4]. Internationally, contributing factors include ageing populations, inadequate social support, and difficulties accessing primary care [2]. Moreover, a growing number of non-urgent patients without immediate life-threatening conditions are managed within the emergency healthcare system [5–8]. Consequently, the role of the EMS has evolved, shifting from primarily transporting patients to the ED to more frequently assessing, treating, and discharging patients on scene, thereby reducing unnecessary admissions [6, 7, 9] and freeing up resources for more urgent cases [10]. This shift has led to a greater emphasis on non-conveyance within EMS systems. However, while over-triage strains scarce resources and challenges the limited capacity of EDs, patient safety must be ensured when deciding to discharge a patient on scene.

The level of non-conveyance varies considerably across countries and regions [11], ranging from 14 to 51% within European EMS [12–15]. However, the patient safety of non-conveyance remains unclear [11], as studies show that within 24–48 h 2.5–6.1% of patients recontact EMS, 4.6–19.0% visit the ED, 1.0–3.3% are hospitalized, and mortality ranges from 0.2 to 3.5% [11]. Moreover, few studies have investigated predictors of recontact with emergency healthcare systems after non-conveyance to determine which patients can safely be discharged at the scene [7, 12, 16–19]. Age is the most commonly reported factor associated with increased risk, impacting both older adults [7, 16–18, 20] and young children [17, 21, 22]. Evidence suggests more frequent recontact after non-conveyance in the night and early morning hours [19, 22, 23]. Also, clinical factors such as hyperglycaemia [19], pain severity, abnormal vital signs [16, 18, 23] and specific conditions like seizures, abdominal pain, and respiratory symptoms are associated with increased risk of recontact with emergency healthcare systems in the current literature [7, 12, 17–19, 22]. However, comparisons between studies are challenging due to variations in methodologies, patient populations, and outcome definitions [11]. Further research is needed to identify patient groups at risk after non-conveyance, as this could inform EMS guidelines and improve patient safety.

This study aimed to investigate the patient safety of EMS non-conveyance by examining proportions of EMS reassessments on-scene, hospital admissions, and mortality within 48 h among non-conveyed patients. Secondarily, we described the proportion of patients within each dispatch code needing reassessments and hospital admission and we explored predictors of these outcomes.

## Methods

### Study design

This register-based study was conducted in the Central Denmark Region, Denmark. We included all non-conveyed patients within the region and followed them for 48 h after their initial emergency call. The reporting follows the Strengthening the Reporting of Observational Studies in Epidemiology (STROBE) guidelines [24].

### Setting

In Denmark, each region is responsible for healthcare services, including EMS [25]. The Central Denmark Region, one of five Danish regions, serves approx. 1.38 million inhabitants. This study includes cases involving medical emergency calls made by patients or bystanders via the national emergency telephone number (1-1-2), as well as calls from general practitioners in primary care to the Emergency Medical Coordination Centre (EMCC) requesting EMS transport. All calls are handled by EMS dispatchers, including nurses and paramedics, with a physician available for consultation [25]. The emergency call is triaged using the Danish Index for Emergency Care [26]. The version used at the time of the study categorized the patient’s primary complaint into one of 37 symptom-based chapters, recommending the appropriate EMS response and urgency level [26]. Urgency levels range from A (potentially life-threatening) to E (handled with phone advice or referral to other services). Lights and sirens are used exclusively for urgency level A, while patients classified as levels B and C must be assessed within 30–120 min [27].

During the study period, 70 ambulances, 2 paramedic-staffed vehicles, and 10 physician-staffed mobile emergency care units (MECU) handled about 130.000 assignments annually [25]. Additionally, a national helicopter emergency medical service (HEMS) with four units covered the entire country [25]. Guidelines allowed EMS providers to treat and discharge patients on scene when hospital transportation was deemed unnecessary. In certain cases, prior consultation with an EMCC physician was mandatory. Thus, non-conveyance could be initiated either at the EMS provider’s discretion or with EMCC physician approval when required (Online Resource 4).

### Data sources

Patient information was routinely recorded in the electronic prehospital medical record. All Danish citizens have a unique personal identification number, which allowed linkage to in-hospital medical records. This enabled us to follow the patient 48-h from the emergency call, identifying any subsequent contact to the emergency healthcare system, and thus ensures prehospital research of high validity [25].

### Study population

All non-conveyed patients attended to by EMS providers in the Central Region Denmark from January 1 st, 2022, to December 31 st, 2023, were included. We defined non-conveyance as a situation where an ambulance or another prehospital unit was dispatched by the EMCC, and the patient was assessed on scene by EMS providers with advanced prehospital competencies, with or without treatment. The EMS providers then made the decision to discharge the patient without transporting them to a healthcare facility. Patients who refused transportation or were transported via other means (e.g. self-transport or taxi) were not included. EMS providers only recommended alternative transportation when it was deemed safe for the patient and aligned with current guidelines. If patients were referred to a taxi by EMS providers, the cost was covered by the EMS. Also, patients with no registration of personal identification number were excluded from the study, as we could not identify subsequent contacts with the healthcare system. Common reasons for patients not having a personal identification number registered include reduced consciousness and confusion. Furthermore, patients were excluded if they were deceased before departure of the EMS unit or received palliative care, in cases where EMS providers concluded that the patient did not require hospital admission.

### Outcomes

Outcomes were EMS provider reassessments, hospital admissions and mortality within 48 h of first emergency call. EMS provider reassessments include all assignments where an ambulance or other prehospital unit was dispatched to the patient again after non-conveyance. Hospital admissions included admissions to the ED or any other hospital department. Patients were followed for 48 h after their first emergency call to allow sufficient time for outcomes to emerge while maintaining a link between the non-conveyance decision and the resulting outcomes. This is in line with several previous studies [7, 12, 13, 19, 23]. Since the time of death was registered only by date, not by time of day, deaths occurring on the same day or within the following 2 days were classified as 48-h mortality.

### Variables

Age and sex were based on patients’ personal identification number. Age was divided into six categories (0–18, 18–34, 35–49, 50–64, 65–79 and ≥ 80 years), while sex was included as a binary variable. Information on urgency level (A-D), time of day (8:00–16:00, 16:00–24:00, 00:00–08:00), EMS on scene time (< 20, 20–34, 35–59, ≥ 60 min) and physician consulted (MECU physician, EMCC physician or no consultation) was extracted from the electronic prehospital medical record. Distance from scene of incident to nearest hospital was calculated for the purpose of the current study, and divided into four categories (< 5 km, 6–20 km, 21–40 km, > 40 km). The EMS dispatch codes assessed by health personnel in the EMCC were aggregated into 16 categories to avoid groups with minimal or no outcomes. Vital signs (systolic blood pressure, body temperature, blood glucose level, heart rate, respiratory rate, oxygen saturation and level of consciousness (Glasgow Coma Scale)) were measured during the prehospital phase. In line with previous research, abnormal vital signs were defined as: systolic blood pressure ≤ 90 mmHg, oxygen saturation < 90%, heat rate ≥ 100 beats per minute, temperature ≥ 37.8 °C, respiratory rate > 24 breaths per minute and blood glucose levels > 14 mmol/L or < 3.5 mmol/L ([16, 28, 29]; See online resource 1). Moreover, for pediatric patients, vital signs were age-adjusted as outlined in the European Resuscitation Council Guidelines (2021) for pediatric life support (See online resource 1 for elaboration). If multiple measurements were made prehospitally, the first measurement was used, as prehospital treatment could have affected later measurements. For patients with missing data, vital signs were classified as normal.

### Statistical analysis

Descriptive data are presented as medians and interquartile range (IQR) for continuous variables, while categorical variables are presented as frequencies (n) and percentages (%), based on non-missing data. To explore possible predictors of EMS reassessment and hospital admission, univariate regression models were performed for each of these two outcomes and possible predicting factors. Log-binomial regression models were used to estimate RRs with 95% confidence intervals (95% CIs). Additional subgroup analyses were conducted focusing on older age groups and vital signs, given their prominence as the most frequently mentioned predictors of recontact with the emergency healthcare system in the existing literature [7, 16–18, 20, 23]. As the underlying assumption of independence of observations could not be fulfilled due to the possibility of multiple EMS assignments deployed to the same patient throughout the data collection period, cluster-robust standard errors were calculated to obtain valid estimation of confidence intervals [30]. Analyses were conducted using StataSE version 18.0 (StataCorp, College Station, TX, USA).

## Results

**Fig. 1:**
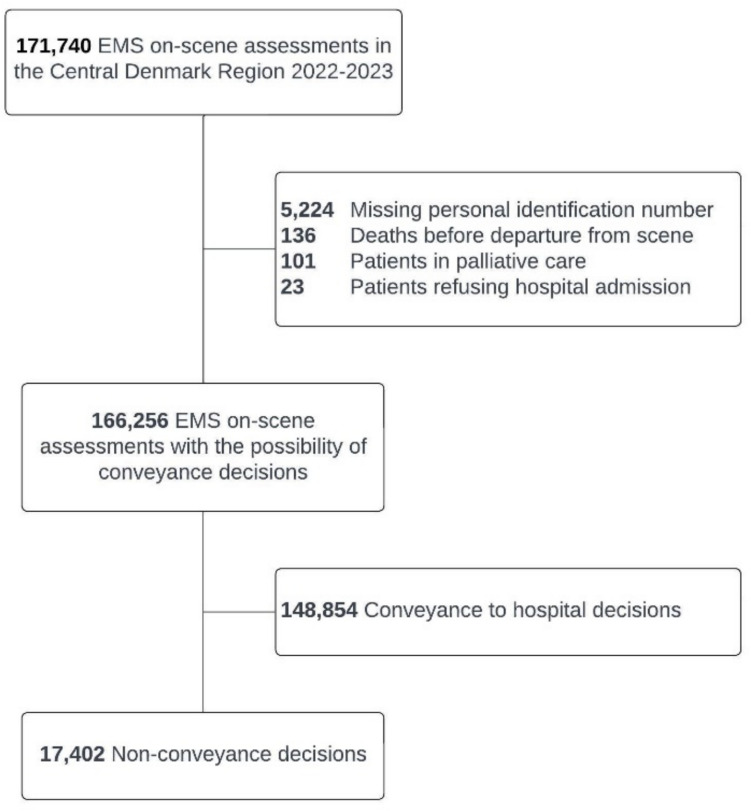
Flowchart. EMS: Emergency Medical Services

**Fig. 2:**
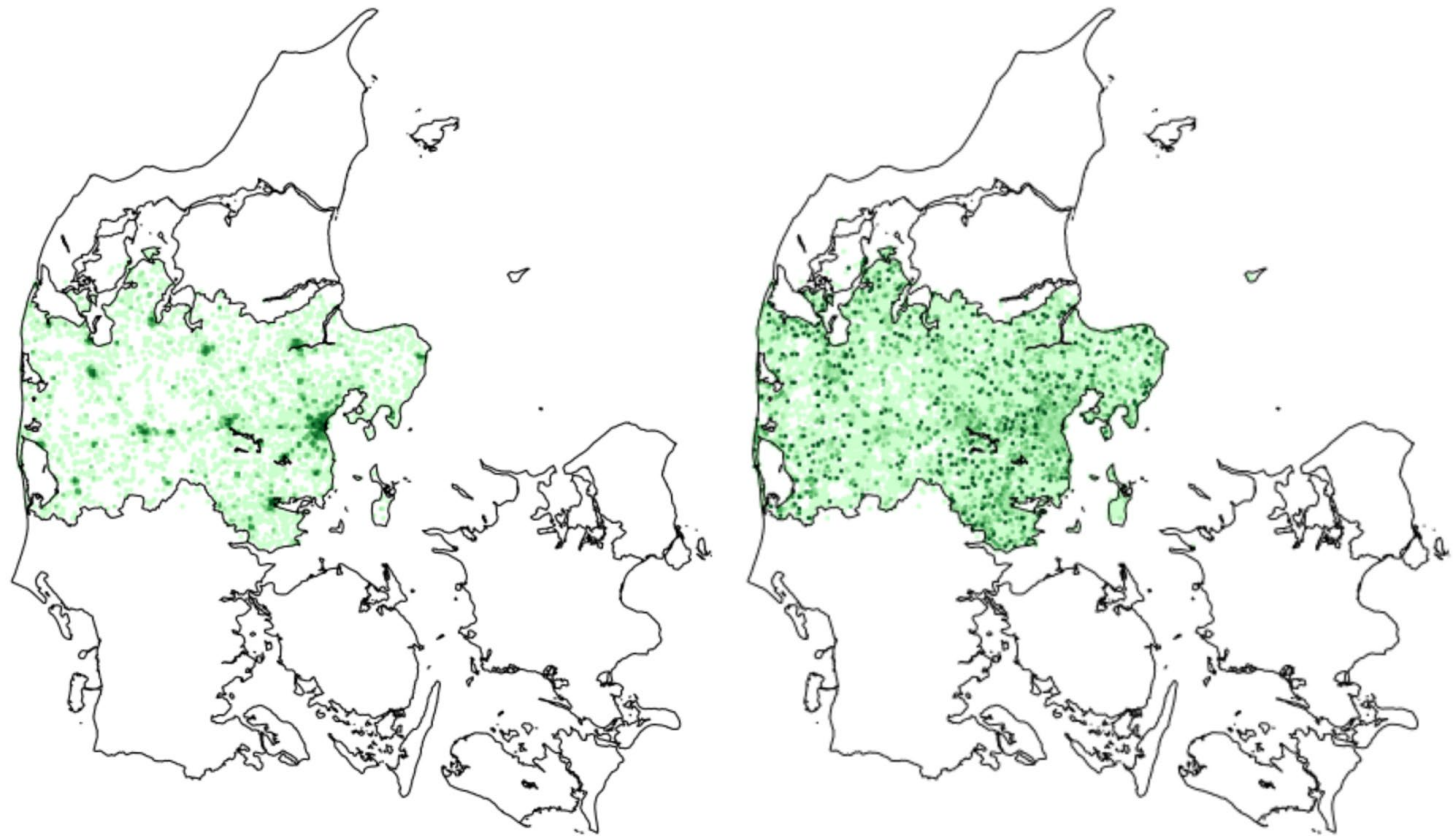
Distribution of non-conveyed patients. The left map displays the absolute numbers of non-conveyed patients while the right map shows non-conveyed patients relative to transported patients (odds). Darker colors indicate higher numbers of non-conveyed patients

During the study period, there were 171.740 emergency calls within the Central Denmark Region in which EMS providers assessed the patient on scene (Fig. 1), ending in either non-conveyance or conveyance to the hospital. After exclusions, 166.256 patients remained, among which 17.402 (10.5%) were non-conveyed. In the non-conveyed group, the sex distribution was equal, and the median age was 53 years (IQR: 27–74) (Table 1). The most common EMS dispatch codes were chest pain (12.1%), impaired consciousness/paralysis (13.9%) and assignments commissioned by a hospital or a physician (16.7%). 52.4% of assignments were dispatched with the highest urgency level, and more than a third of patients (35.7%) had at least one abnormal vital sign in the prehospital phase. EMS personnel spent a median time of 42.4 min (IQR: 31.2–55.2) on the scene before deciding on non-conveyance. Based on absolute numbers, most non-conveyance occurred in areas with larger cities, reflecting population density. However, relative to the number of transported patients, non-conveyance was more evenly distributed throughout the region (Fig. 2).

**Table 1:** Characteristics of EMS assignments ending in non-conveyance (*n* = 17.402)

Variables	
Age, years	
Median (IQR)	53 (27–74)
Missing	0 (0)
Sex	
Male, *n* (%)	8.929 (51.3)
Female, *n* (%)	8.473 (48.7)
Missing, *n* (%)	0 (0)
EMS dispatch code	
Commissioned by hospital/physician, *n* (%)	2.903 (16.7)
Impaired consciousness, paralysis, *n* (%)	2.419 (13.9)
Chest pain, heart disease, *n* (%)	2.103 (12.1)
Accidents, *n* (%)	1.749 (10.1)
Unclear problem, *n* (%)	1.248 (7.2)
Seizure, *n* (%)	1.258 (7.2)
Difficulty in breathing, *n* (%)	1.152 (6.6)
Poisoning, medications, alcohol, drugs, *n* (%)	566 (3.3)
Stomach or back pain, *n* (%)	560 (3.2)
Diabetes mellitus, *n* (%)	458 (2.6)
Minor wound, fracture, injury, *n* (%)	346 (2.0)
Unconscious, *n* (%)	273 (1.6)
Bleeding—non-traumatic, *n* (%)	280 (1.6)
Psychiatry/suicidal ideation, *n* (%)	183 (1.1)
Ear, nose, throat, *n* (%)	131 (0.8)
Other, *n* (%)^a^	1.191 (6.8)
Missing, *n* (%)	582 (3.4)
Level of urgency	
A, *n* (%)	9.121 (52.4)
B, *n* (%)	8.212 (47.2)
C, *n* (%)	65 (0.4)
D, *n* (%)	**–**
Missing, *n* (%)	**–**
Distance to nearest hospital, km	
Median (IQR)	14 (7–28)
Missing, *n* (%)	11 (0.1)
Time of day	
Day (08:00–16:00), *n* (%)	7.285 (41.9)
Evening (16:00–24:00), *n* (%)	6.730 (38.7)
Night (00:00–08:00), *n* (%)	3.387 (19.5)
Missing, *n* (%)	0 (0)
Physician consulted	
No consultation, *n* (%)	2.875 (16.5)
MECU physician, *n* (%)	7.452 (42.8)
EMCC physician, *n* (%)	3.705 (21.3)
Missing, *n* (%)	3.370 (19.4)
EMS on scene time, min	
Median (IQR)	42.4 (31.2–55.2)
Missing, *n* (%)	0 (0)
Abnormal vital signs^b^	
Yes, *n* (%)	6.220 (35.7)
No, *n* (%)	11.182 (64.3)
Missing, *n* (%)^c^	0 (0)
Specific vital signs	
Hypotension (Systolic blood pressure ≤ 90 mmHg or age-adjusted^d^), *n* (%)	193 (1.1)
Tachycardia (Heart rate ≥ 100 beats/minute or age-adjusted^e^), *n* (%)	4.372 (25.1)
Tachypnea (Respiratory rate > 24 breaths/minute or age-adjusted^f^), *n* (%)	690 (4.0)
Desaturation (Oxygen saturation < 90% SpO2), *n* (%)	1.137 (6.5)
Fever (Body temperature ≥ 37.8 °C), *n* (%)	169 (1.0)
Impaired consciousness (Glasgow Coma Scale < 15), *n* (%)	798 (4.6)
Hypo- or hyper-glycaemia (Blood glucose > 14 mml/L or < 3.5 mml/L), *n* (%)	33 (0.2)
Missing, *n* (%)^c^	0 (0)

*IQR* interquartile range, *EMS* emergency medical services

^a^Foreign body in airway, allergic reaction, fire or electricity injury, drowning, animal and insect bites, fever, poisoning in children, childbirth, gynecology/pregnancy, headache, skin and rash, hypo- and hyperthermia, chemicals and gases, eyes, possible death, sick child, urinary system issues, violence/abuse

^b^At least one of the following: Systolic blood pressure, body temperature, blood glucose level, heart rate, respiratory rate, oxygen saturation or level of consciousness (Glasgow Coma Scale)

^c^In our analysis, we assumed that patients with no measurement of vital signs were normal, as the absence of recorded vital signs typically reflects no critical abnormalities observed in EMS practice

^d^Age-adjusted hypotension: 0–1 year: ≤ 50 mmHg, 1–5 years: ≤ 70 mmHg, 5–10 years: ≤ 75 mmHg, 10–18 years: ≤ 80 mmHg

^e^Age-adjusted tachycardia: 0–1 year: ≥ 180, 1–2 years: ≥ 170, 2–5 years: ≥ 160, 5–10 years: ≥ 140, 10–18 years: ≥ 120

^f^Age-adjusted tachypnea: 0–1 year: ≥ 60, 1–2 years: ≥ 50, 2–5 years: ≥ 40, 5–10 years: ≥ 30, 10–18 years: ≥ 25

– = Omitted due to low numbers

### EMS reassessment and hospital admission

Within 48 h of their initial emergency call, 820 patients (4.7%) were reassessed by EMS providers on scene, and 861 patients (4.9%) were admitted to a hospital (Table 2). Among those reassessed by EMS within 48 h, 80.0% were subsequently admitted to the hospital via EMS, predominantly by ambulance. Overall, among all patients admitted to a hospital within 48 h—regardless of whether they were reassessed by EMS—75.7% were transported by EMS, either by ambulance or HEMS.

**Table 2:** Patient outcomes within 48 h of emergency call

Outcome	*n*		*N*	%	95 CI
EMS reassessment	820		17.402	4.70%	4.40–5.03%
Hospital admission		861	17.402	4.92%	4.60–5.25%
Mortality		14	17.402	0.08%	0.04–0.13%

Older age and male sex were both associated with EMS reassessment and hospital admissions by any mode of transport (Table 3). Compared with patients aged 50–64 years (the reference group), the three younger age groups had a markedly lower risk of EMS reassessment and hospital admission within 48 h. The risks for both EMS reassessment and hospital admission were pronounced in patients with dispatch codes for ‘ear, nose and throat’ issues, ‘seizures’, ‘non-traumatic bleeding’, ‘psychiatry/suicidal ideation’ (Table 4). Notably, patients with ‘ear, nose and throat’ complaints had the highest risk of EMS reassessment (17.6%, 95% CI: 11.5–25.2%) and hospital admission (16.0%, 95% CI: 10.2–23.5%). Also, the results tended towards higher risk associated with longer time spent on scene. Surprisingly, no associations were found with urgency level, distance to the nearest hospital, time of day, or physician consultation.

**Table 3:** Patient demographics, clinical presentation, and EMS response characteristics associated with EMS reassessment and hospital admission within 48 h of emergency call among non-conveyed patients (*n* = 17.402)

	EMS reassessment			Hospital admission		
	Yes/N (%)	RR (95% CI)	RD (95% CI)	Yes/N (%)	RR (95% CI)	RD (95% CI)
Age category—*n* (%), years						
< 18	65/2.327 (2.8%)	0.48 (0.34–0.67)	− 3.0 (−4.3;− 1.7)	54/2.327 (2.3%)	0.44 (0.32–0.61)	− 2.9 (− 4.0;− 1.8)
18–34	105/3.568 (2.9%)	0.51 (0.37–0.68)	− 2.9 (− 4.2;− 1.6)	93/3.568 (2.6%)	0.50 (0.37–0.68)	− 2.6 (− 3.8;− 1.5)
35–49	107/2.292 (4.7%)	0.80 (0.58–1.10)	− 1.2 (− 2.8;0.5)	88/2.292 (3.8%)	0.73 (0.53–1.01)	− 1.4 (− 2.8;− 0.1)
50–64	167/2.869 (5.8%)	1.00 (ref)	0.00 (ref)	150/2.869 (5.2%)	1.00 (ref)	0.00 (ref)
65–79	214/3.481 (6.2%)	1.06 (0.82–1.36)	0.3 (− 1.2;1.9)	247/3.481 (7.1%)	1.36 (1.09–1.69)	1.9 (0.5;3.2)
> 80	161/2.865 (5.6%)	0.97 (0.76–1.23)	− 0.2 (− 1.6;1.2)	224/2.865 (7.8%)	1.50 (1.20–1.87)	2.6 (1.2;4.0)
Sex—*n* (%)						
Male	492/8.929 (5.5%)	1.00 (ref)	0.00 (ref)	506/8.929 (5.7%)	1.00 (ref)	0.00 (ref)
Female	337/8.473 (3.9%)	0.70 (0.59–0.83)	− 1.7 (− 2.4;− 0.9)	350/8.473 (4.1%)	0.73 (0.63–0.85)	− 1.5 (− 2.3;− 0.8)
Other^a^	24/1.191 (2.0%)	0.52 (0.34–0.80)	− 1.9 (− 2.9;− 0.8)	27/1.191 (2.3%)	0.52 (0.34–0.78)	− 2.1 (− 3.2;− 1.0)
Level of urgency—*n* (%)						
A	444/9.121 (4.9%)	1.00 (ref)	0.00 (ref)	433/9.121 (4.8%)	1.00 (ref)	0.00 (ref)
B	371/8.212 (4.5%)	0.93 (0.80–1.07)	− 0.3 (− 1.0;0.3)	417/8.212 (5.1%)	417/8.212 (5.1%)	0.3 (− 0.3;1.0)
C	–	–	–	–	–	–
D	–	–	–	–	–	–
Distance to nearest hospital, km						
< 5	201/3.819 (5.3%)	1.00 (ref)	0.00 (ref)	203/3.819 (5.3%)	1.00 (ref)	0.00 (ref)
6–20	288/6.813 (4.2%)	0.80 (0.64–1.01)	− 1.0 (− 2.2;0.1)	296/6.813 (4.3%)	0.82 (0.67–1.00)	− 1.0 (− 2.0;0.0)
21–40	192/4.204 (4.6%)	0.87 (0.67–1.12)	− 0.7 (− 2.0;0.6)	207/4.204 (4.9%)	0.93 (0.75–1.14)	− 0.4 (− 1.5;0.7)
> 40	138/2.555 (5.4%)	1.03 (0.78–1.35)	0.1 (− 1.3;1.6)	149/2.555 (5.8%)	1.10 (0.87–1.38)	0.5 (− 0.8;1.8)
Time of day—*n* (%)						
Day (08:00–16:00)	346/7.285 (4.8%)	1.00 (ref)	0.00 (ref)	374/7.285 (5.1%)	1.00 (ref)	0.00 (ref)
Evening (16:00–24:00)	309/6.730 (4.6%)	0.97 (0.83–1.12)	− 0.2 (− 0.8;0.5)	310/6.730 (4.6%)	0.90 (0.77–1.04)	− 0.5 (− 1.2;0.2)
Night (00:00–08:00)	164/3.387 (4.8%)	1.02 (0.85–1.22)	− 0.2 (− 0.8;0.5)	172/3.387 (5.1%)	0.99 (0.83–1.18)	0.00 (− 1.0;0.8)
Physician consulted—*n* (%)						
No consultation	121/2.875 (4.2%)	1.00 (ref)	0.00 (ref)	128/2.875 (4.5%)	1.00 (ref)	0.00 (ref)
MECU physician	374/7.452 (5.0%)	1.19 (0.96–1.48)	0.8 (− 0.1;1.8)	379/7.452 (5.1%)	1.14 (0.94–1.40)	0.6 (− 0.3;1.6)
EMCC physician	157/3.705 (4.2%)	1.00 (0.80–1.27)	0.00 (− 0.9;1.0)	159/3.705 (4.3%)	0.96 (0.77–1.21)	− 0.2 (− 1.2;0.8)
EMS on scene time						
< 20 min	45/989 (4.6%)	1.00 (ref)	0.00 (ref)	39/989 (3.9%)	1.00 (ref)	0.00 (ref)
20–34 min	157/3.647 (4.3%)	0.95 (0.68–1.31)	− 0.2 (− 1.7;1.2)	157/3.647 (4.3%)	1.09 (0.78–1.54)	0.4 (− 1.0;1.7)
35–59	327/6.976 (4.7%)	1.03 (0.76–1.39)	0.1 (− 1.2;1.5)	327/6.976 (4.7%)	1.19 (0.86–1.65)	0.7 (− 0.6;2.0)
≥ 60 min	132/2.567 (5.1%)	1.13 (0.81–1.57)	0.6 (− 1.0;2.1)	151/2.567 (5.9%)	1.50 (1.06–2.12)	2.0 (0.5;3.5)
Abnormal vital signs^a^—*n* (%)						
No	465/11.182 (4.2%)	1.00 (ref)	0.00 (ref)	475/11.182 (4.3%)	1.00 (ref)	0.00 (ref)
Yes	355/6.220 (5.7%)	1.37 (1.18–1.60)	1.5 (0.8;2.3)	381/6.220 (6.1%)	1.47 (1.28–1.69)	1.9 (1.2;2.6)

*RR* risk ratio, *RD* risk difference, *95% CI* 95% confidence interval, *EMS* emergency medical services, *EMCC* emergency medical coordination centre, *min* minutes

– = Omitted due to low numbers

^a^At least one of the following: systolic blood pressure, body temperature, blood glucose level, heart rate, respiratory rate, oxygen saturation or level of consciousness (Glasgow Coma Scale)

**Table 4:** Proportion of non-conveyed patients within each EMS dispatch code requiring EMS reassessment or hospital admission within 48 h of emergency call (*n* = 17.402)

	EMS reassessment		Hospital admission	
	Yes/N	% (95% CI)	Yes/N	% (95% CI)
EMS dispatch code—*n* (%)				
Commissioned by hospital/physician	113/2.903	3.9% (3.2–4.7%)	127/2.903	4.4% (3.7–5.2%)
Impaired consciousness, paralysis	87/2.419	3.6% (2.9–4.4%)	97/2.419	4.0% (3.3–4.9%)
Chest pain, heart disease	85/2.103	4.0% (3.2–5.0%)	70/2.103	3.3% (2.6–4.2%)
Accidents	49/1.749	2.8% (2.1–3.7%)	69/1.749	3.9% (3.1–5.0%)
Unclear problem	74/1.248	5.9% (4.7–7.4%)	82/1.248	6.6% (5.3–8.1%)
Seizure	114/1.258	9.1% (7.5–10.8%)	99/1.258	7.9% (6.4–9.4%)
Difficulty in breathing	73/1.152	6.3% (5.0–7.9%)	74/1.152	6.4% (5.1–8.0%)
Poisoning, medications, alcohol, drugs	36/566	6.4% (4.5–8.7%)	25/566	4.4% (2.9–6.5%)
Stomach or back pain	25/560	4.5% (2.9–6.5%)	39/560	7.0% (5.0–9.4%)
Diabetes mellitus	23/458	5.0% (3.2–7.4%)	18/458	3.9% (2.3–6.1%)
Minor wound, fracture, injury	17/346	4.9% (2.9–7.8%)	25/346	7.2% (4.7–10.5%)
Unconscious	15/273	5.5% (3.1–8.9%)	17/273	6.2% (3.7–9.8%)
Bleeding—non-traumatic	25/280	8.9% (5.9–12.9%)	26/280	9.3% (6.2–13.3%)
Psychiatry/suicidal ideation	15/183	8.2% (4.7–13.2%)	16/183	8.7% (5.1–13.8%)
Ear, nose, throat	23/131	17.6% (11.5–25.2%)	21/131	16.0% (10.2–23.5%)
Other^a^	24/1.191	2.0% (1.3–3.0%)	27/1.191	2.3% (1.5–3.3%)

*95% CI* 95% confidence interval, *EMS* emergency medical services

^a^Foreign body in airway, allergic reaction, fire or electricity injury, drowning, animal and insect bites, fever, poisoning in children, childbirth, gynecology/pregnancy, headache, skin and rash, hypo- and hyperthermia, chemicals and gases, eyes, possible death, sick child, urinary system issues, violence/abuse

Furthermore, having at least one abnormal vital sign in the prehospital phase was associated with 37% (95% CI: 18–60%) increased risk of EMS reassessment and 47% (95% CI: 28–69%) increased risk of hospital admission. Across the four oldest age-groups, low oxygen saturation (< 90%) was associated with increased risk of EMS reassessment and hospital admission (See online resource 2 and 3). Overall, the highest risk was observed in patients aged 50–64 years with systolic blood pressure below 90 mmHg, for both EMS reassessment (RR: 3.25, 95% CI: 1.55–6.83) and hospital admission (RR: 3.63, 95% CI: 1.73–7.63).

### Mortality

Among all non-conveyed patients who met our inclusion criteria, the 48-h mortality rate was 0.08% (95% CI: 0.04–0.13), corresponding to 14 deaths. Among the excluded patients, a further 260 deaths were recorded, which were unrelated to the non-conveyance decision. These included patients in palliative care (*n* = 101), deaths occurring before EMS providers departed the scene (*n* = 136), and patients who refused treatment (*n* = 23). See Fig. 1.

## Discussion

This study included all non-conveyed patients in the Central Denmark Region during a 2-year period and followed the patients for 48 h after their initial emergency call. Based on our findings, non-conveyance seems to be a safe practice, as most patients did not recontact the emergency healthcare system after being discharged on scene and mortality was low. Among non-conveyed patients, 4.7% (95% CI: 4.4–5.0%) were reassessed by the EMS, while 4.9% (95% CI: 4.6–5.3%) were admitted to the hospital within 48 h. Although it is challenging to determine an acceptable level of recontact that will balance the consideration of resource utilization and patient safety, [31] these findings are comparable to previously published studies from Sweden [20], Finland [19, 32], and Australia [18] with a similar study design. However, differences in follow-up periods, patient populations and definitions of outcomes complicate direct comparisons. These differences may also explain that the mortality was lower in the current study (0.08%, 95 CI: 0.04–0.13%) compared with the general literature (0.2–3.5%), e.g. some previous studies included patient-initiated non-conveyance, which may carry a greater risk of adverse outcomes [11].

While absolute risks were generally low across most patient groups, patient safety can be further enhanced by carefully considering non-conveyance decisions among certain patients with increased risk of reassessment and hospital admission. These includes older patients, males, those with abnormal vital signs and certain EMS dispatch codes. As in previous studies, older patients were more often reassessed by an EMS provider and admitted to the hospital after non-conveyance, when compared to younger patients [7, 16–18, 20]. This could be due to comorbidities, polypharmacy, behavioural changes, and different symptom presentation, contributing to the vulnerability of older patients [16]. More unexpectedly, we also found increased risk of both outcomes among men, when compared with women. Such gender differences have not previously been reported.

In line with previous studies, we found that having one or more abnormal vital signs were predictors of recontact to the emergency healthcare system [16, 18, 19, 23], suggesting that vital signs are helpful indicators of patient risk in relations to non-conveyance, although they should be seen in conjunction with other predictors, especially age and dispatch codes, that were stronger predictors in this study. We carried out additional analyses to gain detailed insights into the association between vital signs and patient outcomes across older age groups. These analyses showed the highest risk among patients aged 50–64 with low systolic blood pressure (< 90 mmHg).

Overall, the highest risk of both EMS reassessment and hospital admission was found among patients with the EMS dispatch code ‘ear, nose, throat’ with 17.6% of assignments ending in EMS reassessment and 16.0% in subsequent hospital admission within 48 h. Hence, EMS providers should critically consider non-conveyance decisions among such patients. Increased risk of both outcomes was also found among non-conveyed patients with the EMS dispatch code ‘seizures’ (e.g. epileptic seizures), which is in accordance with a previous study, where seizures were associated with the highest risk of ED attendance after non-conveyance [17]. The Central Denmark Region guidelines state that patients with seizures can be discharged on scene in cases of known epilepsy if the patient is considered stable after treatment. However, non-conveyance among patients with first-time seizures should be carefully considered. The current study could not differentiate between patients with known epilepsy and first-time seizures but points to greater caution when making non-conveyance decisions among these patients. Furthermore, increased risk was found among patients with psychiatric symptoms, which is in line with previous studies [18, 22]. Some evidence suggests that EMS personnel lack the required skills to manage such patients [33], indicating that new initiatives aimed at reducing the need for subsequent treatment for patients presenting primarily with psychiatric symptoms should be prioritized.

### Strength and limitations

A key limitation of this study was the inability to differentiate between patients only attending the ED within 48 h and those admitted to other hospital departments, as in previous studies [11]. Both outcomes were categorized as hospital admissions, though prolonged admission may indicate a more severe outcome measure than ED attendance only. Additionally, there is potential misclassification in identifying the patient’s primary medical issue, as EMCC personnel assess the main presenting complaint during the emergency call based on brief patient descriptions, often during stressful circumstances. Therefore, the EMS dispatch code may not reflect the final diagnosis. Also, we could not confirm if hospital admission and EMS reassessment were directly related to the non-conveyance decision or a separate medical issue, introducing potential exposure misclassification.

Lastly, we categorized missing measurements of vital signs as normal, as we assumed the EMS personnel measured only relevant vital signs. For instance, most patients did not have their blood glucose measured. We believe that this was due to patients not having any complaints related to hypo- or hyperglycemia. Instead of categorizing the majority of patients with missing vital signs, we found this assumption reasonable for most patients. However, it cannot be ruled out that there was an overrepresentation of missing values among patients with an adverse outcome, hence introducing information bias. Nevertheless, we expect the vast majority of missing values to be attributable to a professional assessment of the patient leading to the conclusion that vital signs were stable.

Notwithstanding these limitations, our study had several strengths. The study had a large sample size and population-based design, including all EMS assignments within the region during a 2-year period. We followed patients for 48 h after first EMS contact by linking their registered personal identification number to in-hospital medical records within the region with less than 7% of assignments lacking such registration. Given that all patients are provided with free healthcare in Denmark, the risk of selection bias is substantially reduced as compared with healthcare system, where financial considerations play an important role in the selection of patients refraining from transportation to the hospital.

### Future directions

Results from the present study can guide EMS providers in making safe non-conveyance decisions on a population-based level. Most importantly, EMS providers should critically consider non-conveyance among patients with ear, nose and throat complaints, and to a lesser extent patients with seizures, non-traumatic bleeding and psychiatric complaints. Although absolute risks were low across most patient groups, patient safety could be further enhanced by carefully considering non-conveyance decisions among older patients and those with abnormal vital signs. Unexpectedly, the risk of both outcomes was higher for men compared with women. Given the exploratory nature of the study, novel findings from the current study should be confirmed by future research before they are integrated into non-conveyance guidelines, including the high risk found among patients with ear, nose and throat complaints. Moreover, we found that EMS providers spend a median 42.4 min (IQR: 31.2–55.2) on-scene, which raises questions of the cost-effectiveness of non-conveyance. While it is beyond the scope of this study, future studies should investigate the cost-effectiveness of non-conveyance, as longer time spend on-scene may warrant a different allocation of resources throughout the healthcare system, if prehospital EMS are increasingly treating and releasing patient on-scene, hence contributing to decreased patient load on EDs. However, releasing the patient on-scene saves the EMS vehicle the added time of transporting and handing over the patient to the hospital, thus freeing up time for new assignments. In Denmark, there is no upper limit on prehospital expenditures. Only in recent years has The Danish Medicines Council been established to assess the value of expensive treatments, however, not at this level.

Furthermore, future studies should aim to develop an algorithm to determine when non-conveyance is safe, preferably based on classification-based methods, like machine-learning, allowing for interpretations on an individual level rather than interpretations on a group-based level, like in the current study. On this basis, we believe our study could be a starting point for identifying relevant predictors that should be validated in future studies.

## Conclusion

Only few non-conveyed patients required reassessment by an EMS provider or were admitted to the hospital within 48 h, while mortality was low, suggesting that non-conveyance is generally a safe practice. However, EMS personnel should critically consider non-conveyance decisions among patients with ‘ear, nose and throat’ complaints, and to a lesser extent patients with complaints related to ‘seizures’, ‘non-traumatic bleeding’ and ‘psychiatry/suicidal ideation’. Also, caution could be applied when considering older patients, males and those with abnormal vital signs. Integrating these considerations into non-conveyance guidelines could help minimize the risk of EMS reassessment and hospital admission and enhance the patient safety of non-conveyance.

## Supplementary Information

Below is the link to the electronic supplementary material.

Supplementary file1 (DOCX 26 KB)

## Data Availability

The datasets analyzed during the current study are available from the corresponding author in anonymized form upon reasonable request but may require completion of a formal data sharing agreement, in compliance with the General Data Protection Regulation (GDPR) and the Central Denmark Region rules.

## References

[1] Andrew E, Nehme Z, Cameron P, Smith K (2020) Drivers of increasing emergency ambulance demand. Prehosp Emerg Care 24(3):385–393 31237460 10.1080/10903127.2019.1635670

[2] Lowthian JA, Cameron PA, Stoelwinder JU, Curtis A, Currell A, Cooke MW et al (2011) Increasing utilisation of emergency ambulances. Aust Health Rev 35(1):63–69 21367333 10.1071/AH09866

[3] Lowthian JA, Curtis AJ, Cameron PA, Stoelwinder JU, Cooke MW, McNeil JJ (2011) Systematic review of trends in emergency department attendances: an Australian perspective. Emerg Med J 28(5):373–377 20961936 10.1136/emj.2010.099226

[4] Christensen EF, Bendtsen MD, Larsen TM, Jensen FB, Lindskou TA, Holdgaard HO et al (2017) Trends in diagnostic patterns and mortality in emergency ambulance service patients in 2007–2014: a population-based cohort study from the North Denmark Region. BMJ Open 7(8):e014508 28827233 10.1136/bmjopen-2016-014508 PMC5724206

[5] Booker MJ, Shaw ARG, Purdy S (2015) Why do patients with ‘primary care sensitive’ problems access ambulance services? A systematic mapping review of the literature. BMJ Open 5(5):e007726-e 10.1136/bmjopen-2015-007726 PMC4442240 25991458

[6] Paulin J, Kurola J, Salanter S, Moen H, Guragain N, Koivisto M et al (2020) Changing role of EMS-analyses of non-conveyed and conveyed patients in Finland. Scand J Trauma Resuscit Emerg Med 28(1):45 10.1186/s13049-020-00741-w PMC7260794 32471460

[7] Todd VF, Swain A, Howie G, Tunnage B, Smith T, Dicker B (2021) Factors associated with emergency medical service reattendance in low acuity patients not transported by ambulance. Prehosp Emerg Care 26(1):66–77 33320722 10.1080/10903127.2020.1862943

[8] Hoikka M, Silfvast T, Ala-Kokko TI (2017) A high proportion of prehospital emergency patients are not transported by ambulance: a retrospective cohort study in Northern Finland. Acta Anaesthesiol Scand 61(5):549–556 28374471 10.1111/aas.12889

[9] Fisher JD, Freeman K, Clarke A, Spurgeon P, Smyth M, Perkins GD et al (2015) Patient safety in ambulance services: a scoping review. Health Serv Deliv Res 3(21):1–250 25996021

[10] Malm F, Elfström A, Ohlsson-Nevo E, Höglund E (2021) Time consumption for non-conveyed patients within emergency medical services (EMS): a one-year prospective descriptive and comparative study in a region of Sweden. PLoS ONE 16(5):e0251686-e 33984054 10.1371/journal.pone.0251686 PMC8118495

[11] Ebben RHA, Vloet LCM, Speijers RF, Tönjes NW, Loef J, Pelgrim T et al (2017) A patient-safety and professional perspective on non-conveyance in ambulance care: a systematic review. Scand J Trauma Resuscit Emerg Med. 10.1186/s13049-017-0409-6 10.1186/s13049-017-0409-6 PMC5513207 28716132

[12] Magnusson C, Herlitz J, Axelsson C (2020) Patient characteristics, triage utilisation, level of care, and outcomes in an unselected adult patient population seen by the emergency medical services: a prospective observational study. BMC Emerg Med 20(1):7 32000684 10.1186/s12873-020-0302-x PMC6993445

[13] Wolthers SA, Mikaelsson TJ, Holgersen MG, Blomberg SNF, Andersen LB, Mikkelsen S et al (2024) Treat and release: an observational study of non-conveyed high-acuity dispatches in a Danish emergency medical system. Intern Emerg Med 19(8):2283–2292 38748389 10.1007/s11739-024-03618-3 PMC11582337

[14] Vloet LCM, Kreek A, Linden EMC, Spijk JJA, Theunissen VAH, Wanrooij M et al (2018) A retrospective comparison between non-conveyed and conveyed patients in ambulance care. Scand J Trauma Resuscit Emerg Med 26(1):91 10.1186/s13049-018-0557-3 PMC6206905 30373652

[15] O’Cathain A, Knowles E, Bishop-Edwards L, Coster J, Crum A, Jacques R et al (2018) Understanding variation in ambulance service non-conveyance rates: a mixed methods study. Health Serv Deliv Res 6(19):1–192 29870196

[16] Lederman J, Lindstrom V, Elmqvist C, Lofvenmark C, Ljunggren G, Djarv T (2021) Non-conveyance of older adult patients and association with subsequent clinical and adverse events after initial assessment by ambulance clinicians: a cohort analysis. BMC Emerg Med 21(1):154 34895152 10.1186/s12873-021-00548-7 PMC8666056

[17] Coster J, O’Cathain A, Jacques R, Crum A, Siriwardena AN, Turner J (2019) Outcomes for patients who contact the emergency ambulance service and are not transported to the emergency department: a data linkage study. Prehosp Emerg Care 23(4):566–577 30582719 10.1080/10903127.2018.1549628

[18] Tohira H, Fatovich D, Williams TA, Bremner AP, Arendts G, Rogers IR et al (2016) Is it appropriate for patients to be discharged at the scene by paramedics? Prehosp Emerg Care 20(4):539–549 26836060 10.3109/10903127.2015.1128028

[19] Laukkanen L, Lahtinen S, Raatiniemi L, Ehrola A, Kaakinen T, Liisanantti J (2022) Emergency department admission and mortality of the non-transported emergency medical service patients: a cohort study from Northern Finland. Emerg Med J 39(6):443–450 33879493 10.1136/emermed-2020-209914

[20] Hoglund E, Schroder A, Andersson-Hagiwara M, Moller M, Ohlsson-Nevo E (2022) Outcomes in patients not conveyed by emergency medical services (EMS): a one-year prospective study. Scand J Trauma Resuscit Emerg Med 30(1):40 10.1186/s13049-022-01023-3 PMC9195370 35698086

[21] Haines CJ, Lutes RE, Blaser M, Christopher NC (2006) Paramedic initiated non-transport of pediatric patients. Prehosp Emerg Care 10(2):213–219 16531379 10.1080/10903120500541308

[22] Oulasvirta J, Salmi H, Kuisma M, Rahiala E, Lääperi M, Harve-Rytsälä H (2019) Outcomes in children evaluated but not transported by ambulance personnel: retrospective cohort study. BMJ Paediatrics Open 3(1):e000523-e 31750406 10.1136/bmjpo-2019-000523 PMC6830473

[23] Nehme E, Nehme Z, Cox S, Smith K (2023) Outcomes of paediatric patients who are not transported to hospital by Emergency Medical Services: a data linkage study. Emerg Med J 40(1):12–19 36202623 10.1136/emermed-2022-212350

[24] von Elm E, Altman DG, Egger M, Pocock SJ, Gøtzsche PC, Vandenbroucke JP (2007) The strengthening the reporting of observational studies in epidemiology (STROBE) statement: guidelines for reporting observational studies. Epidemiology 18(6):800–804 18049194 10.1097/EDE.0b013e3181577654

[25] Lindskou TA, Mikkelsen S, Christensen EF, Hansen PA, Jorgensen G, Hendriksen OM et al (2019) The Danish prehospital emergency healthcare system and research possibilities. Scand J Trauma Resusc Emerg Med. 27(1):100 31684982 10.1186/s13049-019-0676-5 PMC6829955

[26] Andersen MS, Johnsen SP, Sorensen JN, Jepsen SB, Hansen JB, Christensen EF (2013) Implementing a nationwide criteria-based emergency medical dispatch system: a register-based follow-up study. Scand J Trauma Resus Emerg Med 21(1):53 10.1186/1757-7241-21-53 PMC3708811 23835246

[27] Valentin JB, Hansen NH, Behrndtz AB, Væggemose U, Gude MF (2024) Effect of urgency level on prehospital emergency transport times: a natural experiment. Intern Emerg Med 19(2):445–453 38123903 10.1007/s11739-023-03501-7 PMC10954969

[28] Nguyen OK, Makam AN, Clark C, Zhang S, Xie B, Velasco F et al (2017) Vital signs are still vital: instability on discharge and the risk of post-discharge adverse outcomes. J Gen Intern Med 32(1):42–48 27503438 10.1007/s11606-016-3826-8 PMC5215152

[29] Durant E, Sporer KA (2011) Characteristics of patients with an abnormal glasgow coma scale score in the prehospital setting. West J Emerg Med 12(1):30–36 21691469 PMC3088371

[30] Stata. Estimation and postestimation commands. www.stata.com/manuals/u20.pdf

[31] Magnusson C, Hagiwara MA, Norberg-Boysen G, Kauppi W, Herlitz J, Axelsson C et al (2022) Suboptimal prehospital decision- making for referral to alternative levels of care—frequency, measurement, acceptance rate and room for improvement. BMC Emerg Med 22(1):89 35606694 10.1186/s12873-022-00643-3 PMC9125920

[32] Paulin J, Kurola J, Koivisto M, Iirola T (2021) EMS non-conveyance: a safe practice to decrease ED crowding or a threat to patient safety? BMC Emerg Med 21(1):115 34627138 10.1186/s12873-021-00508-1 PMC8502399

[33] Roggenkamp R, Andrew E, Nehme Z, Cox S, Smith K (2018) Descriptive analysis of mental health-related presentations to emergency medical services. Prehosp Emerg Care 22(4):399–405 29364746 10.1080/10903127.2017.1399181

