# Supplementary material for: Patient safety in non-conveyance within prehospital emergency medical services: a register-based study
Source: Intern Emerg Med. 2025 May 22;21(2):673–83. doi: 10.1007/s11739-025-03980-w (PMC13061808; doi:10.1007/s11739-025-03980-w)
Supplement: Supplementary file 1 — Supplementary file1 (DOCX 26 KB) [file 11739_2025_3980_MOESM1_ESM.docx]

## **Supplementary Material**

**Online resource 1:** Cut-off points for abnormal vital signs.

| **Table 5: Definitions of abnormal vital signs** | | | | |  |
| --- | --- | --- | --- | --- | --- |
| **Vital sign** | |  |  | **Cut-off point** | |
|  | Systolic blood pressure | | | ≤ 90 mmHg | |
|  | Heart rate | |  | ≥ 100 beats per minute | |
|  | Respiratory rate | |  | > 24 breaths per minute | |
|  | Oxygen saturation | |  | < 90% SpO2 |  |
|  | Body temperature | |  | ≥ 37.8 °C |  |
|  | Glasgow Coma Scale | | | < 15 |  |
|  | Blood glucose | |  | > 14 mml/L or <3.5 mml/L | |

mmHg: millimeter of mercury; SpO2: saturation of peripheral oxygen; C: Celsius; mmol/L: millimole/Liters

| **Table 6: Definitions of abnormal vital signs among paediatric patients based on guidelines from European Resuscitation Council (34)** | | | | | | | |
| --- | --- | --- | --- | --- | --- | --- | --- |
| **Vital sign** | |  | **< 1 year*** | **1-5 years** | **5-10 years** | **10-18 years** | |
|  | Systolic blood pressure (mmHg) | | ≤ 50 | ≤ 70 | ≤ 75 | ≤ 80 | |
|  |  | |  |  |  | |  |
|  |  | | **< 1 year*** | **1-2 years** | **2-5 years** | **5-10 years** | **10-18 years** |
|  | Heart rate (beats/min) | | ≥ 180 | ≥ 170 | ≥ 160 | ≥ 140 | ≥ 120 |
|  | Respiratory rate (breaths/min) | | ≥ 60 | ≥ 50 | ≥ 40 | ≥ 30 | ≥ 25 |

* Cut-off points for 1 month used for children between 0 and 1 years.

| Online resource 2: Association between abnormal vital signs and EMS reassessment among older age-groups  Table 7 Vital signs and age-groups associated with EMS reassessment among non-conveyance patients (n=17.402) | | | | | | | | | | |
| --- | --- | --- | --- | --- | --- | --- | --- | --- | --- | --- |
|  | | |  | | | | |  | | |
|  |  | **35-49 years** | |  | **50-64 years** |  | **65-79 years** | |  | **80+ years** |
|  |  |  | |  |  |  |  | |  |  |
|  |  | RR (95% CI) | |  | RR (95% CI) |  | RR (95% CI) | |  | RR (95% CI) |
|  |  |  | |  |  |  |  | |  |  |
| Normal systolic blood pressure |  | 1.00 (ref) | |  | 1.00 (ref) |  | 1.00 (ref) | |  | 1.00 (ref) |
| Low systolic blood pressure low |  | - | |  | 3.25 (1.55 - 6.83) |  | 1.79 (0.77 - 4.17) | |  | 1.67 (0.71 - 3.92) |
|  |  |  | |  |  |  |  | |  |  |
| Normal oxygen saturation |  | 1.00 (ref) | |  | 1.00 (ref) |  | 1.00 (ref) | |  | 1.00 (ref) |
| Low oxygen saturation |  | 2.61 (1.31 - 5.17) | |  | 1.72 (1.02 - 2.92) |  | 2.07 (1.47 - 2.93) | |  | 1.49 (0.99 - 2.23) |
|  |  |  | |  |  |  |  | |  |  |
| Normal heart rate |  | 1.00 (ref) | |  | 1.00 (ref) |  | 1.00 (ref) | |  | 1.00 (ref) |
| High heart rate |  | 2.16 (1.41 - 3.30) | |  | 1.26 (0.90 - 1.77) |  | 1.87 (1.24 - 2.80) | |  | 1.33 (0.88 - 2.01) |
|  |  |  | |  |  |  |  | |  |  |
| Normal respiratory rate |  | 1.00 (ref) | |  | 1.00 (ref) |  | 1.00 (ref) | |  | 1.00 (ref) |
| High respiratory rate |  | 1.31 (0.47 - 3.64) | |  | 1.67 (0.77 - 3.64) |  | 2.10 (1.20 - 3.68) | |  | 1.32 (0.73 - 2.38) |
|  |  |  | |  |  |  |  | |  |  |
| Normal Glasgow Coma Scale |  | 1.00 (ref) | |  | 1.00 (ref) |  | 1.00 (ref) | |  | 1.00 (ref) |
| Abnormal Glasgow Coma Scale |  | 2.15 (1.03 - 4.49) | |  | 1.31 (0.64 - 2.65) |  | 1.15 (0.65 - 2.02) | |  | 0.94 (0.47 - 1.87) |

Measurements of body temperature and blood glucose omitted due to a limited number of outcomes within each age group.

- = Omitted due to less than 5 patients with the outcome.

| Online resource 3: Association between abnormal vital signs and hospital admission among older age-groups  Table 8 Vital signs and age-groups associated with hospital admission among non-conveyance patients (n=17.402) | | | | | | | | | | |
| --- | --- | --- | --- | --- | --- | --- | --- | --- | --- | --- |
|  | | |  | | | | |  | | |
|  |  | **35-49 years** | |  | **50-64 years** |  | **65-79 years** | |  | **80+ years** |
|  |  |  | |  |  |  |  | |  |  |
|  |  | RR (95% CI) | |  | RR (95% CI) |  | RR (95% CI) | |  | RR (95% CI) |
|  |  |  | |  |  |  |  | |  |  |
| Normal systolic blood pressure |  | 1.00 (ref) | |  | 1.00 (ref) |  | 1.00 (ref) | |  | 1.00 (ref) |
| Low systolic blood pressure low |  | - | |  | 3.63 (1.73 - 7.63) |  | 2.18 (1.09 - 4.36) | |  | 1.43 (0.66 - 3.10) |
|  |  |  | |  |  |  |  | |  |  |
| Normal oxygen saturation |  | 1.00 (ref) | |  | 1.00 (ref) |  | 1.00 (ref) | |  | 1.00 (ref) |
| Low oxygen saturation |  | 2.45 (1.14 - 5.28) | |  | 1.62 (0.89 - 2.92) |  | 2.08 (1.50 - 2.89) | |  | 1.35 (0.95 - 1.92) |
|  |  |  | |  |  |  |  | |  |  |
| Normal heart rate |  | 1.00 (ref) | |  | 1.00 (ref) |  | 1.00 (ref) | |  | 1.00 (ref) |
| High heart rate |  | 2.07 (1.33 - 3.23) | |  | 1.66 (1.20 - 2.31) |  | 2.07 (1.57 - 2.76) | |  | 1.37 (0.97 - 1.92) |
|  |  |  | |  |  |  |  | |  |  |
| Normal respiratory rate |  | 1.00 (ref) | |  | 1.00 (ref) |  | 1.00 (ref) | |  | 1.00 (ref) |
| High respiratory rate |  | 1.32 (0.49 - 3.56) | |  | 2.14 (1.02 - 4.46) |  | 2.06 (1.27 - 3.32) | |  | 1.65 (1.02 - 2.67) |
|  |  |  | |  |  |  |  | |  |  |
| Normal Glasgow Coma Scale |  | 1.00 (ref) | |  | 1.00 (ref) |  | 1.00 (ref) | |  | 1.00 (ref) |
| Abnormal Glasgow Coma Scale |  | 2.67 (1.50 - 4.76) | |  | 1.46 (0.70 - 3.05) |  | 1.08 (0.62 - 1.86) | |  | 0.90 (0.51 - 1.58) |

Measurements of body temperature and blood glucose omitted due to a limited number of outcomes within each age group.

- = Omitted due to less than 5 patients with the outcome.

Online Resource 4: Table from the prehospital Standard Operating Procedure (SOP) for non-conveyance.

| Situation | Action |
| --- | --- |
| Obvious misunderstanding, where there is no patient in need of treatment. | The case is closed without further action. |
| Competent patient who does not wish to be conveyed to the hospital, and:  - The patient has not received pre-hospital medical treatment, and  - The patient's condition is not considered life-threatening or likely to result in serious disability. | The patient’s wish is respected, and assessment is concluded on-site. Consultation with the on-duty physician in the EMCC is not necessary. |
| Patient does not wish to be conveyed to the hospital, and one or more of the following conditions are present:  - The patient is not competent.  - The patient has received prehospital medical treatment.  - The patient's condition is considered life-threatening or likely to result in serious disability. | The patient must be consulted with the on-duty physician in the EMCC to determine the optimal course of action. |
| Children < 2 years old | The patient must be consulted with the on-duty physician in the EMCC to determine the optimal course of action.  This applies even when the required treatment falls within the competencies of the ambulance personnel or paramedic. |
| Emergency call initiated EMS provider visits where the patient’s condition is assessed, based on the best professional judgment, not to require hospital treatment. It is not crucial whether pre-hospital medical treatment has been provided or not.  Note that there are exceptions to this general principle in the categories below. | The patient must be consulted with the on-duty physician in the EMCC to determine the optimal course of action. |
| Prehospital EMS assessment performed by paramedic vehicles or ambulances with urgency level A or B, as ordered by a general practitioner, out-of-hours primary care (OOH-PC), or staff from the out-of-hours night emergency service (OOH-NES), where the patient has not been seen by a physician or response vehicle within the last 24 hours, and where the patient's condition is clinically assessed as not requiring hospital treatment (it is not essential whether prehospital medical treatment has been provided).  In such cases, the dispatch text include: "Telephone referral and request by doctor (GP)." | The patient must be consulted with the on-duty physician in the EMCC to determine the optimal course of action.  This applies even when the required treatment falls within the competencies of the ambulance personnel or paramedic. |
| Patient seen by their own doctor, OOH-PC, OOH-NES, or emergency team (nurses) within the last 24 hours regardless of the patient's condition.  In such cases, the dispatch text include: "Seen and requested by doctor". | The patient cannot be non-conveyed. |
| Patient assessed at home by ambulance as ordered by the OOH-NES.  In such cases, the dispatch text include: Transport type S-Acute. | The patient must be consulted with the on-duty physician in the EMCC to determine the optimal course of action - whether the patient can be non-conveyed. |
| Children < 1 year old with fever | The patient must be consulted with the on-duty physician in the EMCC.  See the SOP “Fever in Infants” (applies to the entire Prehospital EMS). |

GP = General practitioner, OOH-PC: out-of-hours primary care, OOH-NES: out-of-hours night emergency service
